# Detection of Off-Flavor in Catfish Using a Conducting Polymer Electronic-Nose Technology

**DOI:** 10.3390/s131215968

**Published:** 2013-11-25

**Authors:** Alphus D. Wilson, Charisse S. Oberle, Daniel F. Oberle

**Affiliations:** 1 USDA Forest Service, Southern Research Station, Center for Bottomland Hardwoods Research, Southern Hardwoods Laboratory, P.O. Box 227, Stoneville, MS 38776, USA; E-Mail: coberle@fs.fed.us; 2 USDA Agricultural Research Service, Warmwater Aquaculture Research Unit, Thad Cochran National Warmwater Aquaculture Center, P. O. Box 38, Stoneville, MS 38776, USA; E-Mail: danny.oberle@ars.usda.gov

**Keywords:** artificial olfaction, electronic aroma detection, fish meat quality, volatile organic compounds

## Abstract

The Aromascan A32S conducting polymer electronic nose was evaluated for the capability of detecting the presence of off-flavor malodorous compounds in catfish meat fillets to assess meat quality for potential merchantability. Sensor array outputs indicated that the aroma profiles of good-flavor (on-flavor) and off-flavor fillets were strongly different as confirmed by a Principal Component Analysis (PCA) and a Quality Factor value (QF > 7.9) indicating a significant difference at (P < 0.05). The A32S e-nose effectively discriminated between good-flavor and off-flavor catfish at high levels of accuracy (>90%) and with relatively low rates (≤5%) of unknown or indecisive determinations in three trials. This A32S e-nose instrument also was capable of detecting the incidence of mild off-flavor in fillets at levels lower than the threshold of human olfactory detection. Potential applications of e-nose technologies for pre- and post-harvest management of production and meat-quality downgrade problems associated with catfish off-flavor are discussed.

## 1. Introduction

The occurrence of off-flavor and off-odor in catfish meat is the most economically important problem affecting commercial catfish production in the southern United States [1]. Most pre-harvest and post-harvest off-flavor problems are caused by the presence of malodorous compounds, produced by naturally-occurring aquatic microorganisms (primarily blue-green and actinomycete-type bacteria) during the months of July to September, which are released into the water and then absorbed through the gills, skin or gastrointestinal tract of catfish [2]. These common off-flavor compounds impart a bad flavor and odor to catfish meat, often described as earthy, muddy, or musty to the taste, resulting in reduced flavor quality and a significant reduction in the commercial value (grade) of catfish meat. In many cases, off-flavor catfish meat is unpalatable and unmarketable. Since 1997, channel catfish (*Ictalurus puncatus*) has ranked as the most important pond-cultured catfish species raised for commercial production in the Southern U.S. in terms of total weight sold annually [3]. The incidence of off-flavor in channel catfish, produced during peak harvest months, can account for up to 70% of all harvestable fillets rejected by processors in this region [4].

The current method for detecting off-flavor in catfish meat usually involves quality control evaluations by expensive human inspectors (assessors). The training of assessors for sensory evaluation is necessary for almost all sensory (sight, taste, and smell) methods used to grade meat quality [5]. The degree and costs of training depends on the difficulty and complexity of the assessment. For example, the training of assessors for a large range of meat sample types requires more extensive training in the use of meat-grade scoring systems which vary with different meat types being graded. Sensory quality control also is done by experienced wholesale buyers at the fish market or at quality inspection sites. In addition, sensory assessment often involves taste tests of cooked fish to detect off-flavor and tainted meats. All of these steps in the meat-grading process are time-consuming and involve considerable expense that must be added to the price of the final meat product. A potentially far cheaper, more rapid and efficient method of quality-control screening of fish meats for the presence of off-flavor is to utilize electronic aroma-detection devices, such as electronic noses, that are not subject to human operator fatigue [6–9].

Most previous studies involving the e-nose evaluation of meat quality have been concerned with the detection of microbial and chemical contamination (particularly the presence of human pathogens and toxins in meat), meat age (freshness), spoilage, and authenticity of meat types to avoid fraud and adulterations with cheaper meats [7,10–19]. Electronic-nose devices have been used to assess meat quality in seafood products including fish species such as *Mallotus villosus* (capelin) [20], *Merluccius hubbsi* (Argentinean hake) [21], *Thunnus albacares* (yellowfin tuna) [22], *Salmo salar* (Atlantic salmon) [23,24], *Gadus morhua* (Atlantic cod) [25], *Sardina pilchardus* (Moroccan sardines) [26–29], *Sparus aurata* (sea bream) [30], and *Trichiurus lepturus* (hairtail or cutlassfish) [31]. Meat quality also has been assessed using metal oxide semiconductor (MOS) e-noses for pork [32] and octopus [33]. Previous studies have tested the capabilities of e-nose devices to assess meat quality in only a few freshwater fish species including *Hypophthalmichthys molitrix* (silver carp) [34], *Oreochromis niloticus* (tilapia) [35], and *Ctenopharyngodon idellus* (grass carp) [36].

We evaluated the efficacy of utilizing a conducting polymer (CP) electronic-nose (e-nose) device for the detection of off-flavor in catfish as a quality control for grading meat. The objectives of this study were to determine the capabilities of the Aromascan A32S e-nose: (1) to detect the presence of off-flavor compounds in catfish meat; (2) to discriminate between good-flavor (on-flavor) and off-flavor meat samples; and (3) to assess the potential feasibility of using this or similar e-nose instruments for managing pre-harvest off-flavor problems in catfish ponds and for post-harvest assessments of catfish meat quality and merchantability based on the presence of off-flavor compounds in harvested fish.

## 2. Materials and Methods

The catfish meat samples, utilized for testing the efficacy of the Aromascan A32S e-nose for the detection of off-flavor in this study, were obtained from fresh catfish, randomly collected from several 0.04 ha experimental catfish ponds of the Thad Cochran National Warmwater Aquaculture Center in the Mississippi Delta region located near Stoneville, Mississippi. The fish samples used in this study were all of one species, *Ictalurus punctatus* Rafinesque (channel catfish), the most numerous and abundant catfish species marketed in the southern United States.

### 2.1. AromaScan A32S Electronic Nose

The AromaScan 32S (Osmetech Inc., Wobum, MA, USA) is a conducting polymer (CP) electronic nose that contains an organic matrix-coated polymer-type 32-sensor array, designed for general use applications with 15 v across sensor paths. The sensor-array response of the A32S e-nose to VOCs from different chemical classes was tested and reported previously [37]. Sensors responses were measured as a percentage of electrical resistance changes to current flow in the sensors relative to baseline resistance (%ΔR/R_base_). The sorption of headspace volatiles, composed of specific VOC mixtures, to the conducting polymer sensor surfaces induce a change in the electrical resistance to current flow which is detected by a transducer to produce the output from the sensor array. Sensor responses varied with the type of plastic polymer used in the sensor matrix coating, produced by electropolymerization of either polypyrrole, polyanaline or polythiophene derivatives, which have been modified with ring-substitutions of different functional groups and with the addition of different types of metal ions to the polymer matrix in order to improve and modulate sensor response. All measurements were statistically compared using normalized sensor outputs from the sensor array. The conducting polymer analysis (CPA) methods used with this instrument employ application-specific reference libraries for aroma pattern-recognition and neural-net training algorithms.

### 2.2. Sample Preparation and Prerun Procedures

Fresh catfish (approximately 18 months old and 570–900 g), were randomly sampled by netting from local research ponds after being stunned by a custom built, 40-volt electric pulse shocker (Sylvesters, Inc., Louisville, MS, USA). The heads were removed mechanically using a Baader Model 166 Heading Machine (Baader, Lübeck, Germany), eviscerated manually, filleted and skinned by a Baader Model 184 fillet machine (Baader), and trimmed manually. The fish were immediately frozen at −20 °C for long-term storage prior to e-nose analysis of individual samples. Immediately prior to CPA tests, individual fish were removed from the freezer and thawed to room temperature on clean tissue paper to air dry. Small meat core samples (7 mm-diameter × 2–3 cm-long, depending on fillet thickness) were taken from individual fresh catfish using a sharp stainless steel coring tool, pressed into the side flesh of each fish fillet at the thickest point, and the meat core was removed from the corer using a clean glass rod. Each meat core was blotted on tissue paper to remove free moisture and analyzed separately by placing individual meat cores into a 14.8 cm^3^ uncapped glass vial inserted into a 500 mL Pyrex glass sampling bottle no. 1395 (Corning Inc., Corning, NY, USA) fitted with reference air, sampling, and exhaust ports on a polypropylene bottle cap. Reference air entered the sampling bottle through a 3 mm-diameter polypropylene tube extending to just above the bottom of the sampling bottle. The sampling bottle was held in the sampling chamber within the instrument at a constant air temperature of 25 °C and purged with moisture-conditioned reference air for 2 min prior to building headspace. The sampling bottle was sealed and volatiles from each meat analyte were allowed to build headspace and equilibrate for 30 min prior to each run. Prerun tests were performed as needed to determine sample air relative humidity compared with that of reference air. Reference air was set at 4% relative humidity at 25 °C. The sampling bottle cap and exhaust port were opened between runs to purge the previous sample with conditioned reference air. A reference library (recognition file) for off-flavor *vs.* good flavor meat types was constructed using neural net training by defining aroma classes using reference databases of known sample types. This recognition file then was used to identify unknown samples.

### 2.3. Instrument Configuration and Run Parameters

Electronic-nose analyses of catfish off-flavor compounds in meat were conducted with an Aromascan A32S intrinsically conducting polymer (ICP) e-nose. Eight sensors, (including sensors 11, 12, 21–26, 31 and 32) that did not respond or contribute to the discrimination of catfish volatiles, were turned off. The block temperature was maintained at a constant 30 °C. Reference air was preconditioned by passing room air sequentially through a carbon filter, silica gel beads, inline filter, and Hepa filter to remove organic compounds, moisture, particulates, and microbes, respectively, prior to humidity control and introduction into the sampling bottle. The flow rate of sampled air at the sampling port was maintained at 702 cm^3^/min using a ADM 3000 flow meter (Agilent Technologies, Wilmington, DE, USA). The instrument was interfaced with a personal computer via an RS232 cable controlled with Aromascan Version 3.51 software. The instrument plumbing (reference airflow route through the instrument) was altered from conventional architecture and configured for static sampling of the headspace by allowing air flow, maintained at 605 cm^3^/min flow rate, to be released out of the external vent port of the instrument during analytical runs, and closing the exhaust port on the sampling bottle so that headspace volatiles were taken by suction from a homogeneous static air mass within the sampling bottle.

### 2.4. Data Acquisition Parameters and Run Schedules

Data from the sensor array were collected at 1 s intervals using a 0.2 detection threshold (y-units), a 15–20 y-max graph scale, and with a pattern average of five data samples taken per run during data acquisition. A uniform run schedule was used consisting of reference air 20 s, sampling time 90 s, and wash 20 s, followed by 90 s of reference air for a total run time of 220 s. A 2 min reference air purge was completed between runs after each sample was removed from the sampling bottle. Sensors were rinsed between runs with a 2% isopropanol solution to remove any residual analyte compounds that had adsorbed to the surface of individual sensors from the previous analysis.

### 2.5. Construction of Reference Libraries and Validation

A catfish-specific aroma signature reference library was constructed from ten separate meat samples from separate catfish. All database files were linked to specific (designated) aroma classes defining each sample type or category. All databases were constructed from sensor-array output data collected during a 20 s interval, 85–105 s into the run cycle, immediately prior to the closing of the reference air valve to the sensor array. The following recognition network options (neural net training parameters) were used for each training session: training threshold = 0.60, recognition threshold = 0.60, number of elements allowed in error = 5, learning rate = 0.10, momentum = 0.60, error goal = 0.010 (P = 0.01), hidden nodes = 5, maximum iterations (epochs) = 10,000, using normalized input data, not actual intensity data. Some parameters were modified for improvement of recognition accuracy. A typical training required 2–35 min, depending on the size of the database applied, using an IBM-compatible personal computer with a minimum of 64 Mb of RAM and 350 MHz run speed. Artificial neural net (ANN) trainings were validated by examining training results that compare individual database files for compatibility or by similarity matches to each specific odor classes by test-assigned odor class distributions among related odor classes included in each library. The specific detailed analytical methods used for identification of unknowns, data processing, and statistical determinations followed the procedures and specifications indicated by Wilson *et al.* [37].

### 2.6. Statistical Analysis of Sensor-Output Data

Individual sensor output values from the sensor array were normalized using bell-shaped curve statistical analysis algorithms within the data-processing module of the Aromascan Version 3.51 software. Sensor output values from different samples and replications of each sample type (aroma class) were used to determine mean percentage change in electrical resistance values ± one standard deviation (SD) for each sensor within the aroma signature pattern (aroma profile) of each aroma class.

### 2.7. Principal Component Analysis

Detailed comparisons of relatedness of odor classes (meat types) were determined using principal component analysis (PCA) algorithms provided by the Aromascan 3.51 software. Three-dimensional PCA was used to distinguish between headspace volatiles of good-flavor *vs.* off-flavor catfish meat samples. The mapping parameters for three-dimensional PCA were: iterations = 30, units in Eigen values (%), and use of normalized input data. The degree of relatedness between meat aroma types was determined using three-dimensional PCA of headspace volatiles with each axis (x, y, and z) of the aroma map representing a separate principal component of headspace volatiles within sample-analyte mixtures.

## 3. Results

The utilization of filleted meat core samples from fresh pond-raised catfish provided sufficiently strong sensor-responses from the A32S e-nose sensor array for effective detections of off-flavor compounds in the meat to assess meat quality. The building of sample headspace volatiles from meat samples for 30 min prior to analysis resulted in data outputs that allowed effective discriminations of samples types based on the presence or absence of off-flavor compounds in individual fish samples.

### 3.1. AromaScan A32S E Nose Sensor Array Output

**Figure 1.**
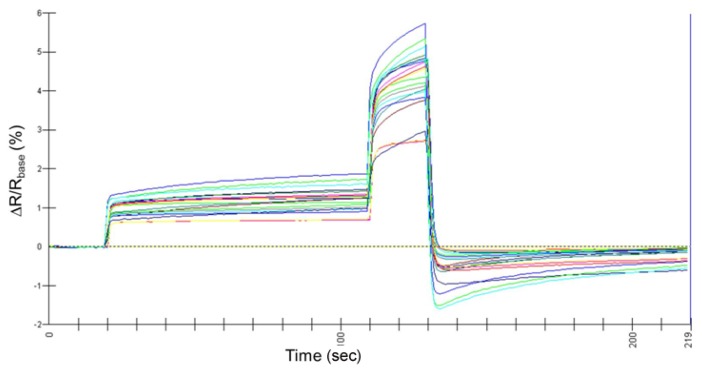
Typical sensor output responses of all twenty sensors in the Aromascan A32S e-nose sensor array to headspace volatiles from catfish meat samples tested for the presence of off-flavor compounds by conductive polymer analyses (CPA). Data values for each graphed line indicate mean percentage change in sensor electrical resistance relative to baseline resistance (ΔR/R_base_%) with each colored line representing a separate sensor output from the sensor array.

The typical graphics outputs from the Aromascan A32S e-nose sensor array, derived from analyses of catfish headspace volatiles, were strong with well-separated intensity readings from individual sensors (Figure 1). All sensor intensity readings during CPA runs were positive relative to baseline readings with no apparent permanent adsorption of headspace analyte compounds to individual sensors. Statistical analysis showed high precision and low variability of individual sensor responses between analytical runs. The wash cycles between analytical runs were effective in preventing carryover of sample analyte compounds between separate analytical runs.

Sensor responses to headspace volatiles from catfish meat samples were quite diffuse, but showed relatively low to moderate intensities. Instrument operation was significantly affected by sample air relative humidity (RH). Sensors were overloaded by excess moisture in the sample. This problem was controlled by maintaining a relatively dry reference air stream (≤4% RH) with minimal impact on sensor sensitivity. Maintaining a low reference air RH assured positive sensor responses in most cases because any additional moisture added to the analyzed headspace was derived from the sample. Effective control of reference air RH by the instrument humidity control device required that sample hydration was properly maintained to avoid free moisture. Standardizing sample preparation and equilibration methods controlled the sample size releasing volatiles and headspace accumulation. Reference air prefilters provided assurances of air quality introduced into the sampling chamber. Instrument precision was very high when these controls were strictly maintained.

### 3.2. Detection and Analysis of Off-Flavor in Catfish

Two distinct aroma classes, good-flavor and off-flavor, were identified from catfish meat samples based on electronic aroma signature patterns (aroma profiles) derived from the A32S e-nose sensor array. Significant differences in sensor-response intensities were found between good-flavor and off-flavor meat samples types (aroma classes) for most sensors used in the analyses (Table 1). Individual sensor responses to good-flavor *vs.* off-flavor aroma classes varied widely within the 2 to 8 sensor-intensity range with very good precision as indicated by low standard deviations (SD) of mean values. Among both sample types, the lowest sensor intensities were recorded for sensors 4–6 and the highest intensities occurred for sensors 27–29. Sensors 30 had no response to headspace volatiles of meats from either aroma class. Sensor 20 could only detect headspace volatiles from off-flavor meat samples. Thus, twenty sensors provided response outputs for both aroma classes.

**Figure 2.**
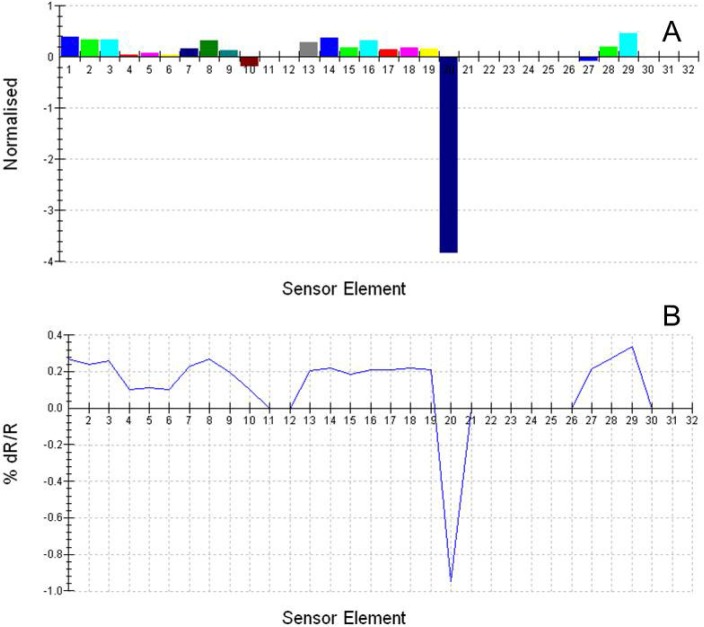
Aroma sensor responses of the A32S sensor array in difference mode. Sensor response percentage differences in e-nose sensor output intensities of individual numbered sensors are indicated for headspace volatiles of good-flavor minus off-flavor catfish meat samples presented as (**A**) bar graph (indicating differences in normalized sensor values) and (**B**) line-graph (indicating percent changes in sensor resistance responses relative to baseline resistance). Sensor element numbers represent individual numbered sensors in the e-nose sensor array.

Comparisons of the sensor-intensity responses of individual sensors in the sensor array were further analyzed using “difference mode” to indicate the intensity differences between good-flavor *vs.* off-flavor sample types, identified as separate aroma classes (Figure 2). Normalized sensor-intensity responses (Figure 2A) were greater than raw sensor-intensity values (Figure 2B) measured as percentage changes in electrical resistance relative to baseline resistance. Positive differences in individual sensor-output intensities, resulting from sensor-output comparisons in “difference mode”, indicated that the sensor recorded a higher intensity for good-flavor meat samples than for the off-flavor meat samples. Negative differences indicated the opposite result in which the sensor recorded a lower intensity for the good-flavor meat samples than for off-flavor samples. Negative differences were recorded for sensors 10, 20, and 27 in the sensor array, but these were rare exceptions in the data. The largest negative difference between good-flavor and off-flavor sensor outputs in “difference mode” occurred for sensor 20 as a result of no output response for this sensor to good-flavor headspace volatiles. All other sensors had positive differences in “difference mode”.

The vast majority (90%) of the differences recorded between good-flavor and off-flavor sensor outputs in “difference mode” were positive for 18 out of 20 sensors producing an output, including sensors 1–9, 13–19, 28, and 29. The percentage difference in sensor intensities for all positive outputs in “difference mode” were less than 0.5%, indicating relatively low variations among individual sensors, although cumulative differences between all twenty sensors provided a high level of overall difference in the aroma signature patterns (profiles) of good-flavor *vs.* off-flavor samples types. The differences for individual sensors outputs, although small, nevertheless are highly significant given that the standard deviations of sensor output responses to headspace volatiles are very low.

Three separate trials (batches) of catfish meat samples were run to test the efficacy of aroma class discrimination between the good-flavor and off-flavor meat sample types. The Aromascan A32S electronic nose provided consistent correct identifications for the majority of the samples tested in three trials based on differences in sensor-array responses to headspace volatiles (Table 2). The instrument correctly identified individual catfish meat cores at rates ranging from 90.7%–98.8% for off-flavor samples and 95.3%–98.5% for good-flavor samples among the three trials. Only one off-flavor sample from trial 3 could not be identified. For these unidentified samples, the ANN algorithm could not assign the aroma profile to a majority aroma class present in the reference library. Other samples that were not conclusively identified, consisting of 20% of the good-flavor samples in trial 2 and 15% of off-flavor samples in trial 3, were classified as indeterminant or inconclusive due to unexplained variations in aroma profile patterns between replications, resulting in only a 65.8% to 67% match with the correct aroma class. Generally, a good statistically valid aroma class determination with good confidence (P < 0.10) requires that an unknown sample has an aroma class match of at least 90% with the aroma elements of a known aroma class in the reference library.

A large proportion (91.4%) of all catfish fillet unknowns from three trials was correctly identified to the proper aroma class. The lowest level (90.7%) of correct identifications that occurred with off-flavor meat samples (in trial 3) were above the statistical level required for a confident determination. All other trial tests for both good-flavor and off-flavor meat types were well above the statistical level necessary for confident determinations. These levels of discriminations were achieved with a sampling rate of ten to twenty replications per unknown meat type in each trial.

### 3.3. Principal Component Analysis

**Figure 3.**
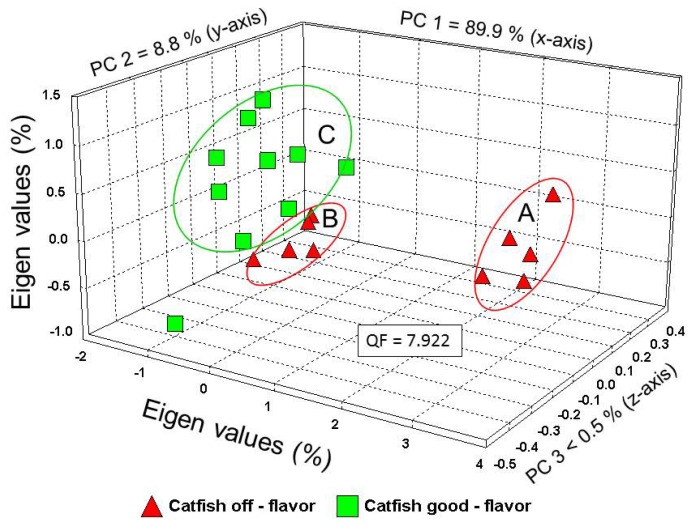
Aroma map of headspace volatiles from good-flavor *vs.* off-flavor catfish meat samples (aroma classes) based on principal component analysis (PCA). The percentages of the total variance, accounting for the variability explained by each orthogonal principal component (PC), are as follows: PC 1 = 88.9%, PC 2 = 8.8%, and PC 3 < 0.5%. The Quality Factor (QF) value of significant difference between the aroma profiles of good-flavor *vs.* off-flavor catfish meat samples was QF = 7.922, indicating a significant statistical difference between these two aroma classes at P < 0.05.

The analysis of headspace volatiles from good-flavor *vs.* off-flavor catfish samples using 3-dimensional PCA indicated significant differences between these two sample groups based on sensor-response patterns (aroma profiles). The differences between the aroma profiles of off-flavor *vs.* good flavor catfish (aroma classes) based on PCA, was graphed in the form of an aroma map (Figure 3).Three separate data clusters were identified on the PCA aroma map for trial 1. Data clusters A and B were clearly identified as belonging to the off-flavor catfish aroma class. Data cluster C was identified as members of the good-flavor catfish aroma class. Samples belonging to data cluster A had a very strong earthy, off-flavor odor, whereas samples in data cluster B exhibited a weak off-flavor aroma. Samples from data cluster C had no noticeable off-flavor aroma that could be easily detected by the human nose. There was only one out-lying good-flavor sample that did not fit within data cluster C. Very similar data-clustering groups and results were found for trials 2 and 3 analyses.

The percentages of the total variance for this analysis, accounting for the variability explained by each orthogonal principal component (PC), were as follows: PC 1 = 89.9%; PC 2 = 8.8%; and PC 3 < 0.5%, representing the x-, y-, and z-axis of the aroma map, respectively. Thus, a high proportion (98.7%) of the variation was explained by the first two principal components (PC 1 and PC 2). Almost all of the data points for individual samples of each aroma class (good-flavor and off-flavor) were closely clustered for most sample replicates on the aroma map.

The statistical level of difference between sensor array output pattern of headspace volatiles from good-flavor *vs.* off-flavor aroma classes were determined using a statistical algorithm called Quality Factor (QF) analysis that determines statistical distances between aroma profiles of aroma classes, measured using Euclidean distance units. The greater the QF value determined from headspace volatiles, the greater the difference (or distance) between the aroma signature profiles of the two aromas being compared. A QF value of 2.0 is roughly equivalent (in statistical terms) to a statistical difference at P = 0.10 level of significance. The QF value determined between the headspace volatiles of the off-flavor *vs.* the good flavor aroma class was 7.92, indicating good discrimination between the two classes at a statistical difference of P < 0.05 level of significance. This QF value indicates that these two aroma classes were distinguished with a high level of confidence and that some of the chemical components of the headspace mixtures of these two aroma classes are not chemically related. Even though cluster groups B (off-flavor) and cluster group C (good-flavor) are in close proximity, there is still sufficient differences among some of the compounds present in the headspace of the off-flavor aroma class that are not present in the good-flavor aroma class.

## 4. Discussion and Conclusions

The Aromascan A32S e-nose provided strong sensor responses to headspace volatiles from fresh catfish meat samples. These results suggest that the time allowed for building headspace volatiles prior to analytical runs might be significantly reduced from 30 min without an appreciable loss of signal strength. Individual sensor outputs derived from CP-analysis of headspace volatiles from catfish core samples were diffuse and well separated, similar to those produced from analyses of headspace volatile metabolites from bacteria, but at lower relative intensities [37]. This divergence of signal outputs from individual sensors suggests the presence of complex mixtures of oxidized polar compounds, such as carboxylic acids and phenolic compounds, characteristic of accumulated metabolic products common in dead animal tissues such as meats [38].

The most likely chemical compounds responsible for differences in aroma profiles between good-flavor and off-flavor catfish aroma classes have been identified previously. The two chemical compounds most frequently associated with off-flavor in catfish meat are geosmin (1,10-*trans*-dimethyl-*trans*-(9)-decanol) and 2-methylisoborneol (MIB) [39,40]. Geosmin and MIB are secondary metabolic products of certain species of bluegreen and filamentous actinomycete-type bacteria (prokaryotes), originally believed to be algae and primitive fungi (eukaryotes) [41]. These compounds are extremely potent and can be tasted in water by humans at concentrations of 0.01 and 0.03 parts per billion (ppb), respectively [4]. The human gustatory (taste) and trigeminal sensory threshold concentrations for the detection of these compounds in channel catfish meat was previously reported as 8.5 ppb for geosmin and 0.8 ppb for MIB [42,43]. The human olfactory (smell) threshold for detection of off-flavor apparently is somewhat higher, precluding the consistent detection of low to moderate levels of off-flavor by trained assessors before processing [41]. This human limitation makes olfactory detection subjective, prone to error, and more difficult to quantify than for more sensitive electronic sensor devices.

Previous studies utilizing electronic-nose devices in catfish meat analyses have been very limited. Korel *et al.* [41] tested the capability of the e-Nose 4000 (EEV Inc., Elmsford, NY, USA) in combination with meat color changes to detect the presence of bacterial spoilage of catfish fillets during cold storage. Most other electronic-nose studies of fish spoilage have involved analysis of various seafood meats to detect the presence of specific types of strong off-odor and off-flavor VOCs resulting from the degradation of particular meat chemical components. Many of these analyses have been described in excellent reviews [5,44]. Total volatile basic amines (TVBAs) are among the most widely used chemical indicators of seafood quality. These include trimethylamine and dimethylamine produced by the autolytic enzymes of spoilage bacteria during frozen storage, ammonia produced by the deamination of amino-acids and nucleotide catabolites, and other volatile basic nitrogenous compounds associated with seafood spoilage [5]. Pacquit *et al.* [45] developed a volatile amine e-nose sensor to monitor fish spoilage. Ammonia is formed by the bacterial degradation/deamination of proteins, peptides and amino acids, and by the autolytic breakdown of adenosine monophosphate (AMP) in chilled seafood products. Ammonia has been found to be an excellent indicator of squid quality [46]. Trimethylamine is a pungent volatile amine associated with the typical “fishy” odor of spoiling seafood and results from the bacterial reduction of trimethylamine oxide (TMAO), naturally present in the living tissues of many marine fish species [5]. The enzyme TMAO dimethylase (TMAO-ase) converts TMAO into equimolar quantities of DMA and formaldehyde (FA). DMA is produced with FA in the meat of fish in the cod (gadoid) family during frozen storage. Dimethylamine is produced autolytically during frozen storage. Fish muscle tissue supports bacterial formation of a wide variety of amine compounds which result from the direct decarboxylation of amino acids. Most spoilage bacteria produce decarboxylase enzymatic activity in response to acidic pH conditions in meats. TVBAs presumably are formed by these spoilage microbes to raise the pH in order to create more favorable conditions for microbial growth. Other volatile amines include histamine, putrescine, cadaverine and tyramine, produced from the decarboxylation of histidine, ornithine, lysine and tyrosine amino acids, respectively. Histamine has received most of the attention since it has been associated with incidents of scombroid poisoning in conjunction with the ingestion of tuna, mackerel, mahi-mahi (dolphin fish) in Hawaii [47–49]. The unsaturated fatty acids found in fish lipids also are highly susceptible to oxidation to produce primarily lipid hydroperoxide oxidation products. The capability of e-noses to detect toxins and other detrimental microbial metabolites in foods provides another useful application for food safety and disease prevention [50,51].

The use of PCA to generate an aroma map of good-flavor *vs.* off-flavor aroma classes revealed three separate data clusters based on differences in aroma profiles with most of the variation represented by only two principal components represented by the x- and y-axes. The occurrence of the strongly off-flavor data cluster A at significant Euclidean distances from the strongly good-flavor data cluster B showed well separation of samples types at the extremes of aroma class sample types. However, the occurrence of data cluster B was interpreted as indications of catfish fillets that contained mostly good flavored aroma elements, but were contaminated by low-levels of off-flavor compounds that rendered the e-nose determination of off-flavor due to sufficient differences generated by the apparent detection of these abnormal malodorous compounds at low concentrations in the meat. Low-level detections of off-flavor compounds in some fillets are viewed as equivalent to incipient detections when off-flavor compounds are just beginning to accumulate in the meat tissue. These data provide evidence that the e-nose has the capability of detecting off-flavor in catfish meat at early stages of contamination. Such samples found in cluster B data were not the same as in-determinant samples because the e-nose clearly distinguished these samples as off-flavored at good levels of statistical confidence.

There are at least two major potential applications of e-nose instruments to help manage and mitigate off-odor and off-flavor meat problems associated with commercial catfish production. First, portable e-nose instruments could be useful to detect and manage the preharvest occurrences of off-flavor in live fish within catfish ponds. The e-nose monitoring of off-flavor incidence in catfish by the periodic random sampling of catfish in individual ponds could be used for the early detection of off-flavor before it builds up to unmanageable levels. Early detection of off-flavor in catfish ponds allows the application of water-management methods to control the build-up of aquatic microbes causing off-flavor before off-flavor compounds began to accumulate within the meat of preharvest catfish. Once the treatment of pond water resources are maintained at sufficient levels of quality, the fish could then be continuously monitored over to time to determine when all traces of off-flavor have disappeared from the fish population in each pond prior to harvest. This process could minimize economic losses to the fish producer by reducing the incidence of catfish off-flavor in the final harvest. Secondly, e-nose devices could be used in the post-harvest meat-production and processing steps for quality control to effectively screen individual fresh catfish fillets for the presence of off-flavor compounds that essentially render affected fish fillets unmerchantable. This screening protocol could facilitate the culling of unsalable meats to assure that only the highest quality product is sold to fish suppliers in commercial meat markets. The use of effective quality assurance and quality control (QA-QC) procedures using e-nose devices could help maintain a producer's high-quality product and associated buyer's brand recognition.

The current study has provided evidence to demonstrate that the CP Aromascan A32S e-nose has the capability to effectively discriminate between good-flavor and off-flavor in catfish fillets. However, the application of similar e-nose instruments to commercial production of catfish fillets would require the use of portable devices with much shorter analysis and recovery times in order to screen fillets with sufficient speed for rapid modern meat-production lines when fish are not prescreened in cultivation ponds prior to harvest. This could be achieved with the use of MOS sensors that not only provide faster recovery times, but also have much longer sensor lives than CP e-nose devices [7]. Once additional research has solved logistic problems associated with these technologies, e-nose instruments ultimately may serve as very effective tools for the screening and quality control (QC) of catfish fillet production. These methods should be equally applicable to other fish species and for screening for other parameters of fish quality controls such as age of meats (shelf-life), presence of human pathogens, contaminations with toxic chemicals, assurance of fish species being sold, and other aroma-based measures of meat quality. The detection of VOCs from biota sources associated with decaying strawberries [52], a fungal biological control agent [53], and components affecting feedstuff quality [54] were reported recently.

## Figures and Tables

**Table 1.** Sensor outputs from the A32S electronic-nose sensor array comparing headspace volatiles released from meat core samples of good-flavor and off-flavor catfish based on conducting polymer (CP) analyses.

	**Sensor Number†**										
Meat type	1	2	3	4	5	6	7	8	9	10	13
Off-flavor	4.99 ± 0.04	4.56 ± 0.04	5.18 ± 0.03	2.80 ± 0.01	2.76 ± 0.01	2.81 ± 0.01	5.80 ± 0.01	5.66 ± 0.01	4.87 ± 0.03	4.85 ± 0.05	4.01 ± 0.01
Good-flavor	5.38 ± 0.01	4.89 ± 0.02	5.52 ± 0.02	2.84 ± 0.01	2.83 ± 0.01	2.84 ± 0.01	5.96 ± 0.01	5.95 ± 0.01	5.00 ± 0.01	4.66 ± 0.01	4.29 ± 0.01

	**Sensor Number†**										

Meat type	14	15	16	17	18	19	20	27	28	29	30
Off-flavor	3.58 ± 0.00	4.25 ± 0.02	3.83 ± 0.01	5.23 ± 0.01	5.33 ± 0.01	5.09 ± 0.01	3.82 ± 0.05	7.40 ± 0.02	6.81 ± 0.03	6.36 ± 0.02	NR
Good-flavor	3.96 ± 0.01	4.42 ± 0.01	4.15 ± 0.01	5.37 ± 0.01	5.51 ± 0.01	5.25 ± 0.01	NR	7.32 ± 0.02	7.00 ± 0.03	6.83 ± 0.03	NR

† Each sensor in the sensor array was coated with a different intrinsically conducting polymer, (composed of polypyrrole, polyanaline, or polythiophene derivatives), modified by proprietary ring-substitutions with different functional groups to impart unique conductive properties (resistance responses to VOCs). All conducting polymers were doped with specific metal ions to improve and modulate polymer conductivity and sensor sensitivity. Values for each sensor are mean normalized data (transformed from raw data of sensor intensities) expressed as mean ΔR/R_base_% ± SD, derived from ten sample replications per sensor type. NR indicates no sensor response was produced or recorded for this meat type (aroma class). All sensor values for off-flavor *vs.* good-flavor meat types (for each sensor) were significantly different at the P < 0.001 level of significance, except for numbered sensors with NR.

**Table 2.** Conducting polymer analysis tests of the capability of the AromaScan A32S e-nose to identify and discriminate between good-flavor *vs.* off-flavor catfish meat cores.

**Trial**	**Meat Type**	**n**	**Correctly Identified ^1^**	**Indeterminant ^2^**	**Unknown Determination ^3^**	**Aroma Class Match ^4^**
1	Off-flavor	10	100.0	0.0	0.0	98.2
	Good-flavor	10	100.0	0.0	0.0	98.5
2	Off-flavor	10	100.0	0.0	0.0	98.8
	Good-flavor	10	80.0	20.0 (65.8)	0.0	95.3
3	Off-flavor	20	80.0	15.0 (67.0)	5.0 (51.2)	90.7
	Good-flavor	10	100.0	0.0	0.0	97.3

1 Percentage of catfish meat samples that were correctly identified.

2 Percentage of unknown catfish meat samples for which a percentage match to a single aroma class was not high enough to make a clear identification. Values in parentheses indicate the mean percentage match determined from among all samples placed into this determination category.

3 Percentage of unknown catfish meat samples for which data from the sensor array could not assign a match to any aroma class. Values in parentheses indicate the mean percentage match determined from among all samples placed into this determination category.

4 Mean percentage match of unknown catfish meat core samples to the correct aroma class identity.
